# Health consequences of paternal incarceration using a future-treated control group

**DOI:** 10.1186/s40352-025-00395-9

**Published:** 2026-01-17

**Authors:** Erin J. McCauley, Camille Portier, Katherine LeMasters

**Affiliations:** 1 Department of Social and Behavioral Sciences, University of California, San Francisco, San Francisco, USA; 2 Philip R. Lee Institute for Health Policy Studies, University of California, San Francisco, San Francisco, USA; 3 Department of Political and Social Sciences, European University Institute, Florence, Italy; 4 Division of General Internal Medicine, University of Colorado Anschutz Medical Campus, Aurora, USA; 5 Department of Epidemiology, University of Colorado Anschutz Medical Campus, Aurora, USA

**Keywords:** Paternal incarceration, Health, Access to health care, Utilization of health care, Health behaviors, Adolescent health

## Abstract

**Background:**

Paternal incarceration is a now a common experience in the life course for young people in the United States that likely shapes health. This study examines the effect of paternal incarceration on adolescents’ health care access/utilization and health behaviors during adolescence. This study employs an innovative approach, which we refer to as the future treated control method, to adjust for unobserved heterogeneity by exploiting plausibly exogenous variation in the timing of paternal incarceration to develop a future treated control group. In this approach youth who have experienced paternal incarceration are compared to youth who will experience paternal incarceration after the outcomes are measured. Results are replicated using a linear probability model with a traditional reference group of those who have not experienced paternal incarceration.

**Results:**

Paternal incarceration is associated with a higher probability of having no insurance (b = 0.06, *p* = 0.04), forgoing needed medical care (b = 0.09, *p* = 0.03), and receiving psychological counseling (b = 0.08, *p* = 0.01) after reducing unobserved heterogeneity. Paternal incarceration is not significantly associated with health behaviors when adjusting for unobserved heterogeneity, despite significant associations between paternal incarceration and alcohol and tobacco use using a traditional regression model. In this case, paternal incarceration has an independent effect on health care access and use in adolescence but not observed health behaviors.

**Conclusions:**

Paternal incarceration has implications for health care use during the transition to adulthood. Paternal incarceration should be considered a distinctive risk factor that contributes to health inequity in the U.S. and research on the collateral consequences of incarceration for families needs to address the threat of selection.

The U.S. has the highest incarceration rate among high income countries; the incarcerated population in the US has increased substantially from just over 500,000 adults in 1980 to over 2.16 million in 2016 (Bureau of Justice Key Statistics, 2018). Parental incarceration has similarly increased over this period, paralleling the rise in mass incarceration in both size and disproportionality (Wildeman, 2009). Recent evidence suggests this trend of mass parental incarceration has continued to grow and concentrate among the most disadvantaged (Enns et al., 2019). Recent estimates from the Family History of Incarceration Survey (FamHIS) indicate that 45% of U.S. adults report having ever had an immediate family member incarcerated and around 20% report having ever had a parent incarcerated (Enns et al., 2019). Moreover, this risk is concentrated among younger cohorts. One in just three Americans between the ages of 18 and 20 report having ever had a parent incarcerated (Enns et al., 2019). The experience of parental incarceration is now a common event in the life course of disadvantaged children and a prominent yet understudied instrument of social and health inequality.

A large body of research has documented associations between parental incarceration and poor health. Parental incarceration is associated with infant and child mortality (Turney, 2017; Turney & Goodsell, 2018; Wildeman et al., 2018; Wildeman, 2012; Wildeman et al., 2014; Yi et al., 2021), mental health difficulties, including depression, migraines, and anxiety, and development delays, such as conduct issues and learning disabilities (Lee et al., 2013; Turney, 2014). However, most research focuses on childhood, with much less known about the consequences during adolescence, a critical developmental period when health behaviors crystalize and health care access becomes increasingly important for managing emerging health concerns (Harris et al., 2006; Umberson et al., 2008). Moreover, most prior studies rely on associational design that cannot address the substantial threat of unobserved selection into parental incarceration (Baker, 2023; McCauley, 2020; Wakefield & Wildeman, 2014). Unobserved selection is a central issue in the study of parental incarceration because families that experience parental incarceration are likely to differ systemically from families that do not experience parental incarceration in difficult-to-measure ways that also shape children’s health and wellbeing.

In this study, we use an innovative method we refer to the as future treated control group approach where we compare youth whose fathers have been incarcerated to those whose fathers will be incarcerated in the future. We aim to build on the ample research demonstrating negative associations between paternal incarceration and child health to strengthen causal inference by exploiting plausibly exogenous timing variation. This approach allows us to better isolate the effect of paternal incarceration net of stable unmeasured characteristics that differentiate families with incarcerated fathers. Using this method, we evaluate whether paternal incarceration affects adolescent health through two theoretically distinct pathways: (1) risky health behaviors, which are likely to be linked to stress and (2) reduced access to health care, which is likely to be linked to economic hardship. Identifying whether parental incarceration operates through health care access, health behaviors, or both is essential for understanding how it contributes to the early emergence of health inequality; in this study, we find evidence for a structural pathway.

## Theoretical mechanisms

Parental incarceration exposes children to significant acute and chronic stressors—including family instability, caregiver strain, and stigma (Turney, 2014); Phillips & Gates, 2011; Wildeman, 2010; Wildeman & Western, 2010). Parental incarceration also shapes children’s exposure to stress by affecting the wellbeing and health of the family left behind, including the children’s caregivers, such as grand-parents and mothers (Goldman, 2019; Lee et al., 2014), and the wellbeing of the incarcerated parent after release (Massoglia & Pridemore, 2015; Schnittker & John, 2007). Stress is therefore a plausible mechanism linking parental incarceration to health, including through its association with risky health behaviors such as smoking tobacco, drinking alcohol, and excessive eating. Individuals experiencing stress may attempt to alleviate it by using these behaviors as a source of relaxation and pleasure (Pampel et al., 2010; Umberson et al., 2008). Engagement in risky health behaviors is more common among children growing up in less stable family structures, and family disruption may have consequences for children’s health behaviors—for example, family instability is associated with smoking (see Kirby, 2002). Consistent with this mechanism, research has found that paternal incarceration is associated with elevated engagement in risky health behaviors. For instance, a review of the literature on parental incarceration and illicit substance use found that 75% of studies reported significant increases in substance use associated with parental incarceration (Rowell-Cunsolo et al., 2024).

A second, distinct mechanisms, involves changes in the structural conditions that support access to health care for adolescents. Burdens associated with parental incarceration are also likely to compromise families’ access to health care and health-enhancing resources (Lee et al., 2014). Parental incarceration is associated with decreased financial resources, during incarceration and after release (Geller et al., 2011; Wakefield & Uggen, 2010). This increased financial strain could affect uptake of health insurance, as well as contribute to families being underinsured where insured families still avoid care due to cost (Galbraith et al., 2005; Wherry et al., 2016). More than a quarter of low-income families with children are underinsured with health care costs which exceed 10% of their income (Galbraith et al., 2005). Indeed, recent work examining the effect of parental incarceration on the oral health of children has found that children of incarcerated parents have worse oral health, are more likely to report unmet dental health needs, and that economic hardship attenuates these relationships (Testa et al., 2020). Research on stigma further suggests that both caregivers and adolescents may avoid interactions with medical institutions due to anticipated judgement or discrimination (Aronson et al., 2013; Penner et al., 2018; Phillips & Gates, 2011). Indeed, research on racial discrimination, stigma, and stereotype threat in the realm of health care utilization evidence a link between perceived stigma and avoidance of routine care and medical interactions (Penner et al., 2018; Thompson et al., 2004). Finally, is also important to move beyond individual behaviors to understand and address health disparities (Braveman & Gottlieb, 2014; Galea & Link, 2013).

These two pathways—one operating through stress-related changes in health behaviors and the other through disrupted access to care—are theoretically separable and carry different policy implications. In this study, however, they function as conceptual frameworks for interpreting the results rather than as mechanisms that we directly observe or formally test.

### Contributions

By leveraging timing-based identification strategy through the use of the future treated control group method, this study moves beyond prior associational research to provide stronger evidence related to how paternal incarceration causally influences adolescent health. In doing so, we provide some evidence of whether the observed associations with health in prior work could reflect behavioral change, reduced access to health care resources, or both. Distinguishing these mechanisms while adjusting for unobserved heterogeneity is critical for understanding how early-life inequalities emerge and for identifying intervention points that can mitigate the intergenerational health consequences of mass incarceration.

## Methods

This paper examines how paternal incarceration shapes health care utilization and health behaviors during adolescence by adjusting for unobserved heterogeneity through the use of a future treated control group. By exploiting plausibly exogenous variation in the timing of paternal incarceration, we create a strategic comparison group comprised of respondents who will be incarcerated after the outcomes are measured.

National Longitudinal Study of Adolescent to Adult Health (AddHealth) is a nationally representative longitudinal survey of adolescents in the United States (*N* = 20,745) (Harris et al., 2009). The survey focuses on adolescent health, health behaviors, personal traits, families, schools, and communities. The first wave was conducted in middle and high schools, and a subsample was selected for the longitudinal sample for home interviews for six waves. This study employs data from the first four waves (Harris et al., 2009). Participants are included in the analytic sample for this study (*n* = 5, 527) if they participated in Wave IV (when paternal incarceration age was collected) and have complete data on the predictors with the exception of household income, which was imputed.

### Key variables

The primary exposure was paternal incarceration. Children who reported only maternal incarceration (*n* = 229) were excluded from the analytic sample (*n* = 5,298) to focus specifically on paternal incarceration. We concentrate on paternal incarceration in this study for two reasons: it is considerably more common and, in these data, occurs sufficiently often to use the methods detailed here. Information about paternal incarceration status and age of the respondent during paternal incarceration was collected retrospectively during Wave IV of the study. We collapse this information to create five categories: (1) paternal incarceration before the respondent’s first birthday (*n* = 16); (2) paternal incarceration between the respondent’s first birthday and Wave I (when the outcomes are measured) (*n* = 372); (3) paternal incarceration at an unknown time (*n* = 194); (4) no paternal incarceration (*n* = 4,594); and (5) paternal incarceration after Wave I (*n* = 122) (Figure A.1). We refer to the category of individuals whose father was incarcerated after Wave I as the “futures” category because they had not experienced the event at the time of outcome data collection but will experience paternal incarceration in the future. Following prior applications of this strategy (e.g., McCauley, 2020; Porter & King, 2015), we retain all categories in the regression model to fully adjust for exposure history and avoid introducing bias. However, only the ‘pre,’ ‘future,’ and ‘never’ categories are used as analytic comparison groups in the identification strategy.

We utilized two sets of dependent variables; health care utilization or access and health behaviors. Outcome variables were collected during Wave I, when participants are between seventh and twelfth grade. For health care access and use, we examine forgoing needed medical care, not having health insurance, having a dental exam in the past year, having a physical exam in the past year, and receiving psychological counseling in the past year. The outcomes related to health behaviors are if the participants have drunk alcohol, if they have smoked cigarettes, if they exercise three or more times a week, and if they ate vegetables or carbohydrates yesterday. All outcomes are dichotomous. When examining the effect of paternal incarceration on smoking and drinking alcohol, we compared the reported age of first cigarette and age of first drinking alcohol to age of paternal incarceration. For those who experienced paternal incarceration prior to Wave I, we excluded participants where there was not sufficient time ordering (i.e. they had smoked a cigarette or drank alcohol before experiencing paternal incarceration). In a sensitivity analysis, we included these individuals, and the results were substantively similar.

All models include family and individual level covariates and longitudinal survey weights to adjust for attrition. The covariates include respondent’s gender, age, race (non-Hispanic White, non-Hispanic Black, Hispanic, and another non-Hispanic race), if the mother reports being in good health (Y/N), mother’s education (less than high school degree, high school degree or equivalent, and more than high school), mother’s marital status (single, married, widowed, divorced, separated), mother’s race (non-Hispanic White, non-Hispanic Black, Hispanic, and non-Hispanic other race), if their mother was born in the U.S. (Y/N), their mother’s age, which census region they reside in, and household income (measured in thousands of dollars). Participants were excluded from the analytic sample if they were missing covariate values, with the exception of household income. We used multiple imputation to addressing missing household income because of the high level of missingness (Rubin, 1987). The imputation was conducted in Stata and used six imputations.

### Analytic strategy

This study uses two model specifications to estimate the effect of paternal incarceration on utilization of health care and health behaviors. First, we use an innovative approach, which we refer to as the future treated control method. In this model, we compare youth who have experienced parental incarceration prior to the measurement of health outcomes at Wave I, to youth who will experience parental incarceration after Wave I (i.e. remove the “nevers” from a traditional reference group so that the reference group is only the “futures”). Those who have experienced paternal incarceration are therefore compared to those that will experience paternal incarceration in the future using a linear probability model. We use a linear probability model so that we can compare the coefficient value between Model 1 and Model 2, consistent with past applications of tis future treated control group (e.g., McCauley, 2020; Porter & King, 2015).

By exploiting plausibly exogenous variation in the timing of paternal incarceration, this method reduces the threat of unobserved heterogeneity in estimating the effect of paternal incarceration. Iterations of this approach, while lesser used, has been successfully employed in the study of the collateral consequences of family incarceration on other domains, such as criminal behaviors (Porter & King, 2015), school outcomes (McCauley, 2020), infant health (Testa et al., 2020), and most recently college expectations and aspirations (Baker, 2023). We refer to our design as the future treated control method to distinguish it from the broader category of “strategic comparison group” approaches. Strategic comparison groups can take many forms, including comparisons on alternative shock or other sources of counterfactual approximation (e.g., Cho, 2009; McCauley, 2021). Our approach relies specifically on exploitation of the timing of treatment, and naming the method explicitly clarifies the unique assumptions and supports reproducibility in future research. However, we want to emphasize that the approach here is in line with that taken by Porter and King (2015), and later McCauley (2020) and Baker (2023).

An important consideration to note is that the future treated control model uses a smaller comparison group (only those who will experience paternal incarceration after the outcomes are measured) which can reduce power and precision, leading got greater probability of null findings. To determine if a loss of significance between Model 1 and Model 2 is likely attributable to the reduced comparison group size rather than the reduction of omitted variable bias, we will use a comparison of the coefficients (Porter & King, 2015). The futures comparison group sizes for each outcome can be found in Table A.1 of the Appendix. Our approach to defining the treated (paternal incarceration between age 1 and W1) and future treated groups (paternal incarceration between WI and WV) follows prior implementations of this design (e.g. McCauley, 2020; Porter & King, 2015). Narrowing the age range of the comparison windows would reduce group sizes and statistical power, and prior work employing narrow age groups (e.g., Baker, 2023) shows that estimates using this strategy are not highlight sensitive to model age group adjustments.

The future treated control method rests on the assumption that families which have experienced and will experience paternal incarceration are more similar on a variety of measured and unmeasured domains than those that have experienced and never will experience paternal incarceration. To investigate this assumption, we compare the means between the intervention group (paternal incarceration prior to Wave 1) and the two comparison groups (never and futures) (Table 1). The descriptive pattern suggests the intervention and futures group are more similar than the intervention and never group. This assumption is further investigated in the Appendix through formal difference tests (Table A.2) providing additional support that the assumption is plausibly met. Finally, we note that because the future-treated group consists of adolescents who will experience paternal incarceration after Wave I, our timing-based comparison identifies the effect of paternal incarceration among those who are ever treated within the observation window. This yields an average treatment effect on the treated, or ATT-type estimand, rather than a full-population average treatment effect (ATE). Our estimates using the future treated control approach therefore describe the effect of paternal incarceration for adolescents who are on a trajectory of eventually experiencing it.

**Table 1:** Mean values and proportions for key variables among parental incarceration groups of interest

			Parental Incarceration Group Means		
			Pre W1	“Futures”	“Nevers”
**Predictors**					
	Race				
		NH-White	0.48	0.53	0.61
		NH-Black	0.27	0.23	0.15
		Hispanic	0.18	0.15	0.14
		NH-Other	0.07	0.10	0.09
	Male		0.42	0.49	0.45
	Age		15.48	15.04	15.51
	Family Income		35.53	42.44	52.83
	Mother’s Age		40.48	39.55	42.31
	Mother’s Health		0.82	0.88	0.89
	Mother US Born		0.89	0.83	0.83
	Mother’s Race				
		White	0.51	0.53	0.64
		Black	0.26	0.21	0.15
		Hispanic	0.17	0.18	0.13
		Other non-white	0.05	0.09	0.09
	Mother’s marital status				
		Single	0.10	0.06	0.03
		Married	0.53	0.64	0.80
		Widowed	0.04	0.00	0.03
		Divorced	0.24	0.24	0.11
		Separated	0.09	0.06	0.03
	Mother’s Education				
		Less than HS	0.22	0.20	0.14
		HS or equivalent	0.32	0.30	0.28
		More than HS	0.46	0.50	0.58
	Regions				
		West	0.27	0.24	0.24
		Midwest	0.24	0.25	0.35
		Northeast	0.10	0.10	0.14
		South	0.39	0.54	0.39
Outcomes					
	Health care Access				
		Forgone medical care	0.25	0.21	0.18
		No Insurance	0.12	0.08	0.06
		Dental Exam	0.60	0.68	0.72
		Physical Exam	0.62	0.63	0.66
		Psychiatric Exam	0.17	0.11	0.11
	Health Behaviors				
		Drink	0.62	0.56	0.55
		Smoke	0.65	0.56	0.54
		Exercise 3 + times a week	0.55	0.58	0.52
		Ate carbohydrates yesterday	0.91	0.93	0.93
		Ate vegetables yesterday	0.62	0.63	0.69

*N* = 5,298. NH is non-Hispanic, US is United States, HS is high school

Then, we estimate the effect on each outcome using a traditional linear probability model (LPM) comparing youth who experienced paternal incarceration prior to Wave I to youth who have not experienced paternal incarceration by Wave I (i.e. the reference groups includes the “nevers” and “futures” categories combined).

## Results

7% (*n* = 372) of the sample experienced paternal incarceration prior to Wave I (Table 2). The sample is 46% male and nearly three-fourths of the sample is non-Hispanic White (74%). 88% of participant’s mothers reported good or excellent health, and half had more than a high school degree (55%). Nearly 80% of mothers were married, and 90% were born in the U.S. The average age when the outcomes were collected is age 15.

**Table 2:** Demographic information about the sample (Add Health, *N* = 5,298)

Variables		Proportion/ Mean (SD)	*n*
Paternal Incarceration			
	Before Age 1	< 0.01	16
	Before Wave 1	0.07	372
	Timing unknown	0.04	194
	Nevers	0.87	4594
	Futures	0.02	122
Male		0.46	
Age Wave I		15.28 (1.76)	
Race			
	NH-White	0.74	
	NH-Black	0.10	
	Hispanic	0.10	
	NH-other	0.06	
Mother in Good Health		0.88	
Mother’s Education			
	Less than HS	0.13	
	HS	0.31	
	More than HS	0.55	
Mother’s Martial Status			
	Single	0.03	
	Married	0.79	
	Widowed	0.03	
	Divorced	0.12	
	Separated	0.04	
Mother’s Race			
	NH-White	0.76	
	NH-Black	0.10	
	Hispanic	0.09	
	NH-other	0.05	
Mother is US born		0.90	
Mother’s Age		41.74 (6.05)	
Household Income		50.93 (51.05)	
Region			
	West	0.18	
	Midwest	0.28	
	Northeast	0.13	
	South	0.40	
Health care Access			
	Forgone medical care	0.17	
	No Insurance	0.07	
	Dental Exam	0.73	
	Physical Exam	0.66	
	Psychiatric Exam	0.12	
Health Behaviors			
	Drink	0.54	
	Smoke	0.56	
	Exercise 3 + times a week	0.51	
	Ate carbohydrates yesterday	0.94	
	Ate vegetables yesterday	0.70	

NH is non-Hispanic, US is United States, HS is high school. Standard deviation (SD) is in parenthesis when mean is reported

### Health care access and utilization

In Table 3, Model 1 employs the future treated control group as the reference. We find that paternal incarceration is, on average, associated with a nine-percentage point higher probability of foregoing needed medical care (*p* = 0.03) and a six-percentage point higher probability of not having health insurance (*p* = 0.04). Paternal incarceration is not significantly associated with the probability of having a physical exam (*p* = 0.29) or dental exam in the past year (*p* = 0.22). Last, paternal incarceration is associated with an eight-percentage point higher probability of receiving psychological counseling (*p* = 0.01).

**Table 3:** Linear probability models estimating the association between paternal incarceration and health care access and utilization using the future treated reference group and a traditional reference group using addhealth data (1994–2019)

	Forgone Medical Care	No Health Insurance	Physical Exam	Dental Exam	Psychological Counseling
Model 1: Futures Reference Group	0.09* (0.04)	0.06* (0.03)	-0.05 (0.05)	-0.06 (0.05)	0.08* (0.03)
Model 2: Traditional Reference Group	0.07*** (0.02)	0.06*** (0.02)	-0.03 (0.03)	-0.08** (0.02)	0.07*** (0.02)
*N*	5,293	4,733	5,286	5,283	5,292

Model 1 uses reference group of “futures”; Model 2 uses reference group of combined “futures” and “nevers”. Sample size varies due to missingness on the outcome variable. All models include control variables for participant gender, age, race, mother’s health, mother’s education, mother’s marital status, mother’s race, if mother was born in the United States, mother’s age, household income, and census region. All control variables are measured at Wave 1 and longitudinal survey weights are used. *p<0.05, **p<0.01, ***p<0.001

We can explore the role of unobserved heterogeneity in the association between paternal incarceration and health care access by comparing these results to the traditional regression which does not adjust for unobserved heterogeneity (Model 2). We see a similar pattern, with paternal incarceration being significantly associated with the probability of forgoing medical care (*p* < 0.001), reporting no health insurance (*p* < 0.001), and receiving psychological counseling (*p* < 0.001), but not being significantly associated with the probability of having a physical exam (*p* = 0.21). Paternal incarceration is not significantly associated with the probability of reporting a dental exam in the past year (*p* = 0.22) when using the future treated control group, Model 1, but is significantly associated with a lower probability of reporting a dental exam using the traditional reference group in Model 2 (*p* < 0.01). This suggests that the effect seen in Model 2 is possibly due to unobserved heterogeneity, and when compared to a more similarly disadvantaged group children of incarcerated fathers are not significantly less likely to access dental care.

### Health behaviors

Using the future treated control as the reference group, paternal incarceration is not associated with health behavior outcomes, as seen in Table 4. Paternal incarceration is not significantly associated with the probability of drinking alcohol (*p* = 0.38), smoking cigarettes (*p* = 0.26), exercising three or more times a week (*p* = 0.13), eating carbohydrates (*p* = 0.52), or eating vegetables (*p* = 0.11). However, using a traditional regression in Model 2 paternal incarceration is associated with a seven-percentage point higher probability of drinking alcohol (*p* = 0.01) and a nine-percentage point higher probability of having smoked a cigarette (*p* < 0.00). While the comparison group for Model 1 is smaller, introducing the possibility that the difference in significance between the models is a result of differing comparison group size, the coefficients are sufficiently different to suggest that the differences may result from the better adjustment for unobserved heterogeneity.

**Table 4:** Linear probability models estimating the association between paternal incarceration and health behaviors using the future control reference group and a traditional reference group using addhealth data (1994–2019)

	Health Behaviors				
	Drink	Smoke tobacco	Exercise 3 + times a week	Ate carbohydrates yesterday	Ate vegetables yesterday
Model 1: Futures Reference Group	0.04 (0.05)	0.06 (0.05)	-0.08 (0.05)	-0.02 (0.03)	0.08 (0.05)
Model 2: Traditional Reference Group	0.07* (0.03)	0.09** (0.03)	-0.00 (0.03)	0.01 (0.01)	-0.01 (0.03)
*N*	5,258	5,225	5,295	5,292	5,295

Model 1 uses reference group of “futures”, Model 2 uses reference group of combined “futures” and “nevers”. Sample size varies due to missingness on the outcome variable. All models include control variables for participant gender, age, race, mother’s health, mother’s education, mother's marital status, mother's race, if mother was born in the United States, mother's age, household income, and census region. All control variables are measured at Wave 1 and longitudinal survey weights are used. Individuals who experience parental incarceration after they begin smoking or drinking are excluded from those respective analyses due to time ordering. *p<0.05, **p<0.01

## Discussion

In this study, we investigate the association between paternal incarceration and adolescents’ health care utilization and health behaviors using both an innovative approach that employs a future treated control group as the reference for paternal incarceration and a traditional regression model. We find that paternal incarceration is associated with lower access to health care (including higher probabilities of foregone medical care and reporting no health insurance) and a higher probability of psychological counseling. We also find that paternal incarceration has no association with health behaviors when controlling for unobserved heterogeneity, but that there is a significant positive association for substance use using a traditional regression model.

This study finds that the effects of paternal incarceration and health care access are mixed. While paternal incarceration is associated with an increased probability of forgoing needed medical care and lacking health insurance, paternal incarceration is positively associated with receiving psychological counseling. Although the prevalence of psychological counseling remains low overall, this increase suggests that some children affected by paternal incarceration are being connected to needed services. The stress and trauma of paternal separation is likely a key mechanism behind the deleterious consequences of paternal incarceration on children (Geller et al., 2010; Turney, 2014), indicating the increased probability of psychological counseling—though limited—is promising. We hypothesize two pathways which could lead to this increased access to counseling; (1) youth could be connected to psychological services specifically through institutions working as a social safety net (such as school based health centers providing counseling), or (2) the sigma of parental incarceration could contribute to youth being involved in school discipline as a pathway towards receiving mandated psychological services (for example see: Wildeman et al., 2017). Future research should explore these potential pathways, as they would likely have different consequences for children’s health. Additionally, there is no significant effect of paternal incarceration on the probability of receiving a physical exam in either model. Two-thirds of participants had received a physical exam, so this may also be a result of schools or other institutions providing some routine care to all children.

Although children of incarcerated parents have some access to care, these youth are still forgoing needed medical care and have a lower probability of having health insurance compared to youth who have not and will not experience paternal incarceration and compared to those who will experience paternal incarceration in the future. These results are in line with the conceptualization of paternal incarceration as a unique childhood risk factor for health (Lee & Wildeman, 2021; Wakefield & Wildeman, 2014; Wildeman et al., 2018). Beyond the loss of financial resources and unstable family households, paternal incarceration acts as an additional disadvantage on health care access. One potential pathway contributing to this result could be stigma, which might prevent youth and their families from interacting with the medical profession and other institutional agents because they are aware of the bias and discrimination they may experience (Phillips & Gates, 2011; Wildeman et al., 2017). Forced single parenthood through the incarceration of a child’s father may make logistics of seeking care and signing up for government provided medical insurance more difficult, or families could be engaging in institutional avoidance (Dwyer Emory & Sementilli, 2025 ; Haskins & Jacobsen, 2017).

There are implications of this work for the long-term consequences of paternal incarceration for health over the life course and for the well documented disparities in health that children with incarcerated parents face (Wildeman et al., 2018). Beginning to understand health in the early life course is crucial to understanding health trajectories (Harris et al., 2006). Childhood disadvantage is associated with poor health behaviors and health outcomes, and set youth on life trajectories marked by disadvantage, impacting their health and socioeconomic outcomes in adulthood (Ferraro et al., 2016). Paternal incarceration is thus an added dimension of disadvantage through decreased health care access and use for youth. Moreover, paternal incarceration is disproportionally experienced by those who are already more likely to be disadvantaged such as Black individuals or children living in poverty (Wildeman, 2009). This added childhood disadvantage is thus likely to further perpetuate health disparities.

When considering health behaviors, the results of this study suggest that paternal incarceration is not independently associated with health behaviors. While a significant effect of paternal incarceration on substance use was found with a traditional regression model, this association was not significant using the future treated control method to adjust for unobserved heterogeneity. This suggests that unobserved heterogeneity may play a role in observed associations between paternal incarceration and youth substance use. Factors such as stress stemming from overall disadvantage, social network negative influence, or lack of community resources could confound the relationship between paternal incarceration and health behaviors, which are better accounted for by having a more similar comparison group. However, the future treated control group regression may bias results towards the null due to the differing cell sizes between the models. Given volume of research finding that parental incarceration is associated with elevated rates of substance use in the existing research (Rowell-Cunsolo et al., 2024), future research employing other methods to adjust for unobserved heterogeneity, such as fixed effect approaches or an instrumental variable approach, should explore parental incarceration and the onset of substance use to confirm our results. It is also important to note that we only consider the onset of substance use not continuation or chronicity. While important to consider, future research should also examine a broader variety of substances and consider chronicity of substance use.

Finally, this work has methodological contributions. This study provides further evidence that exploiting plausibly exogenous variation in the timing of paternal incarceration to develop a future treated control group is a useful approach (Baker, 2023; McCauley, 2020; Porter & King, 2015). The differing results between this innovative approach and the traditional regression model also underscores the importance of adjusting for unobserved heterogeneity when studying the effect of paternal incarceration. This is consistent with existing studies finding that the effect of parental incarceration for some outcomes maybe explained, at least in part, by selection (e.g., McCauley, 2020; Wildeman & Turney, 2014).

### Limitations

In this study, we were unable to examine heterogeneity of results by race or socioeconomic status. The comparison group sizes are smaller when using the future treated control group approach, thereby limiting our ability to parse these data into smaller groups. However, the risk of parental incarceration varies by race and socio-economic status (Enns et al., 2019; Wildeman, 2009) and prior work has found heterogeneity in the effect of parental incarceration on children (Turney & Wildeman, 2015). Moreover, race and socioeconomic status are already associated with differential access to health care and health behaviors during adolescence (Hanson & Chen, 2007; Keyes et al., 2015; Spencer & Grace, 2016). Future research should explore potential heterogeneity in the associations between parental incarceration and access to health care and between parental incarceration and health behaviors during adolescence.

There are limitations regarding the Add Health data source. First, it that it relies on self-reported data, including paternal incarceration, health behaviors, and health care access, which may be inaccurately reported. Additionally, the measure of paternal incarceration is retrospective and therefore may be inaccurately reported in the case of youth who misremember their age during paternal incarceration and may be underreported in the case of youth who forget or are unaware of early life paternal incarceration or whose parents hide the incarceration to protect their child. One way that we minimize this bias is by collapsing the age of paternal incarceration into categories, including a category for those who do not remember the timing of paternal incarceration. Collapsing the age categories minimize the effect of misremembering the timing of paternal incarceration in early life, because all paternal incarceration between birth and Wave I (when participants were between 7th and 12th grade) is collapsed into one category. By middle school, youth would be able to rely on their own memory instead of others retellings of paternal incarceration when participants were too young to remember themselves (Bryan, 2017). Third, the Add Health data, like all longitudinal survey data, likely under samples justice-involved families (Western & Pettit, 2005, 2010). This concern, while less pressing in the context of our study which estimates within-sample associations using a timing-based approach, is important regardless and should be considered a limitation of this work. Another important limitation is that there was substantially more missingness for one variable—household income. To address this issue and avoid a loss of power, we used multiple imputation for this variable. Future studies should use other approaches.

Finally, while the future treated control group method aims to *reduce* the effect of unobserved heterogeneity in estimation it is not a panacea. There could be additional unobserved factors between families that do and do not experience parental incarceration which we are unable to capture. Our approach rests on the assumption that paternal incarceration timing during a child’s life after age one is plausibly exogenous. Prior work shows that criminal offending declines sharply beginning during pregnancy, with substantial desistance in the first year of parenthood (Massenkoff & Rose, 2024). Our analytic design deliberately avoids comparing paternal incarceration groups across this sensitive period of pregnancy and childbirth. We limit comparisons to two groups whose paternal incarcerations occur after age 1: those who experience paternal incarceration between ages 1 and Wave I and those who experience paternal incarceration after Wave I. By this point, parents have already passed through the major desistance “turning point” associated with pregnancy and childbirth (Massenkoff & Rose, 2024). Thus, the relevant identifying assumption is not that paternal incarceration timing is exogenous across the life course, but rather that the specific timing of paternal incarceration in childhood through early adulthood is not systematically correlated with unobserved predictors of children’s health, conditional on extensive covariates capturing parental background, SES, early family circumstances, and pre-treatment child characteristics. However, to the extent any residual nonrandom timing exists, it would likely attenuate estimated differences, yielding conservative estimates of the impact of paternal incarceration.

## Conclusion

Incarceration, in addition to being a social determinant of health for individuals, acts as an intergenerational social determinant of health through shaping access to health care for children of incarcerated parents. Paternal incarceration is a risk factor that contributes to health inequity in the U.S. by differentiating access to and utilization of health care during adolescence. While the social safety net may help these youth to seek some care, including psychological counseling, children of incarcerated parents remain disadvantaged in terms of health care access relative to their peers. Public health researchers should examine why youth who have experienced paternal incarceration forego needed medical care and lack health insurance, and the pathways through which these youth are accessing physical exams and more psychological counseling than their peers to better understand how to similarly expand access to other care. Furthermore, as youth with incarcerated fathers are no more likely than equally disadvantaged youth to participate in most risky health behaviors, health care practitioners should focus on interventions targeting access to and use of health care instead of focusing on individual health behaviors. Adolescence is a crucial period in the life course for health as it sets individuals on health trajectories, making the potential of expanded access to care during this pivotal period critical for health equity.

## Figures and Tables

**Fig. 1:**
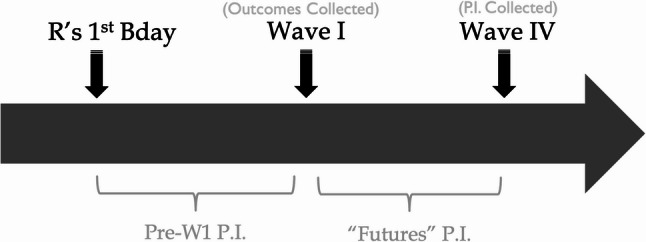
Timeline of data collection and the analytic comparison in the future treated control method

## Data Availability

No datasets were generated or analysed during the current study.

## Appendix

**Table 5:** Table A.1 Cell sizes for respondents who experienced paternal incarceration prior to the outcomes being measured in W1 (“PreW1”) and those who experienced paternal incarceration after the outcomes were measured (“futures) which are compared in Models 2 of Table 3 and Table 4

Outcomes	Prior Parental Incarceration	Future Parental Incarceration
Health care Access		
Forgone medical care	97	19
No Insurance	40	7
Dental Exam	221	82
Physical Exam	224	79
Psychiatric Exam	72	13
Health Behaviors		
Drink	225	60
Smoke	256	68
Exercise 3+ times a week	190	73
Ate carbohydrates	116	344
Ate vegetables/fruit	235	70
N	372	122

**Table 6:** Table A.2. Difference tests for key variables between parental incarceration groups to test if families which have experienced parental incarceration (Pre-W1) are more similar to those who will experience paternal incarceration after the outcomes are measured (“futures”) than those who never experience parental incarceration during data collection (“Nevers”)

	Parental Incarceration Group Means			P-Value from Difference Tests	
	Pre W1	"Futures"	"Nevers"	Pre vs. Futures	Pre vs. Nevers
Predictors					
Race					
NH-White	0.48	0.53	0.61	0.19	0.00
NH-Black	0.27	0.23	0.15	0.17	0.00
Hispanic	0.18	0.15	0.14	0.25	0.01
NH-Other	0.07	0.10	0.09	0.14	0.04
Male	0.42	0.49	0.45	0.06	0.21
Age	15.48	15.04	15.51	0.00	0.73
Family Income	35.53	42.44	52.83	0.06	0.00
Mother’s Age	40.48	39.55	42.31	0.13	0.00
Mother’s Health	0.82	0.88	0.89	0.03	0.00
Mother US Born	0.89	0.83	0.83	0.03	0.00
Mother’s Race					
White	0.51	0.53	0.64	0.70	0.00
Black	0.26	0.21	0.15	0.14	0.00
Hispanic	0.17	0.18	0.13	0.88	0.00
Other non-white	0.05	0.09	0.09	0.10	0.01
Mother’s marital status					
Single	0.10	0.06	0.03	0.08	0.00
Married	0.53	0.64	0.80	0.00	0.00
Widowed	0.04	0.00	0.03	0.00	0.03
Divorced	0.24	0.24	0.11	0.92	0.00
Separated	0.09	0.06	0.03	0.20	0.00
Mother’s Education					
Less than HS	0.22	0.20	0.14	0.57	0.00
HS or equivalent	0.32	0.30	0.28	0.62	0.03
More than HS	0.46	0.50	0.58	0.35	0.00
Regions					
West	0.27	0.24	0.24	0.39	0.06
Midwest	0.24	0.25	0.35	0.95	0.66
Northeast	0.10	0.10	0.14	0.79	0.00
South	0.39	0.54	0.39	0.38	0.99

Outcomes					
Health care Access					
Forgone medical care	0.25	0.21	0.18	0.27	0.00
No Insurance	0.12	0.08	0.06	0.23	0.00
Dental Exam	0.60	0.68	0.72	0.04	0.00
Physical Exam	0.62	0.63	0.66	0.84	0.01
Psychiatric Exam	0.17	0.11	0.11	0.05	0.00
Health Behaviors					
Drink	0.62	0.56	0.55	0.10	0.00
Smoke	0.65	0.56	0.54	0.01	0.00
Exercise 3+ times a week	0.55	0.58	0.52	0.43	0.13
Ate carbohydrates yesterday	0.91	0.93	0.93	0.54	0.03
Ate vegetables yesterday	0.62	0.63	0.69	0.84	0.00

*N*=5,298. NH is non-Hispanic, US is United States, HS is high school
